# Etymologia: *Orthobunyavirus*

**DOI:** 10.3201/eid2205.ET2205

**Published:** 2016-05

**Keywords:** etymologia, Orthobunyavirus, Bunyaviridae, Inkoo virus, Chatanga virus, viruses

## *Orthobunyavirus* [orʺtho-bunʹyə-viʺrəs]

The largest genus in the family *Bunyaviridae*, the genus *Orthobunyavirus* was originally named *Bunyavirus*, for the type species Bunyamwera virus, first isolated in 1943 from the eponymous town in western Uganda. Originally, the vernacular term “bunyavirus” was used for viruses in this genus, but as more genera were added to *Bunyaviridae* (there are currently 5), confusion arose over whether “bunyavirus” referred to members of the genus *Bunyavirus* or family *Bunyaviridae*.

In 1995, the Bunyaviridae Study Group of the International Committee on Taxonomy of Viruses recommended adding the prefix “ortho-” (Greek for “correct”) to the genus name (C. Calisher, pers. comm.) to prevent confusion. Two orthobunyaviruses reported on in this issue of *Emerging Infectious Diseases* are Inkoo virus and Chatanga virus (named for the towns of Inkoo, Finland, and Khatanga, Russia, respectively, where they were first isolated).
